# Measuring person-centered integrated care for people living with mild to moderate chronic kidney disease and multimorbidity: a cross-sectional survey

**DOI:** 10.3389/frhs.2025.1655472

**Published:** 2025-09-18

**Authors:** Taylor Hecker, Sabrina Jassemi, Liza van Vliet, Nancy Verdin, Nazret Russon, Meghan J. Elliott, Brenda R. Hemmelgarn, Maria J. Santana, Kimberly Manalili, Kerry McBrien, Aminu K. Bello, Amity Quinn, Pim Valentijn, Maoliosa Donald

**Affiliations:** ^1^ Department of Community Health Sciences, Cumming School of Medicine, University of Calgary, Calgary, AB, Canada; ^2^ Essenburgh Research & Consultancy, Essenburgh Group, Harderwijk, Netherlands; ^3^ Patient Partner, University of Calgary, Calgary, AB, Canada; ^4^ Department of Medicine, University of Alberta, Edmonton, AB, Canada; ^5^ Department of Family Medicine, University of Calgary, Calgary, AB, Canada; ^6^ Hanze University of Applied Sciences, Groningen, Netherlands

**Keywords:** chronic kidney disease, multimorbidity, person-centered integrated care, multidisciplinary care, patient-oriented research

## Abstract

**Introduction:**

Person-centered integrated care (PC-IC) has been shown to improve health outcomes for individuals with chronic conditions. However, there is limited evidence measuring PC-IC delivery to people with mild to moderate chronic kidney disease and co-morbidities. We aimed to assess PC-IC delivery for this population in Alberta, Canada.

**Methods:**

We conducted a survey (May-December 2023) using the Rainbow Model of Integrated Care Measurement Tool via weblink or telephone to quantify PC-IC using a 5-point Likert agreement scale. Patients with chronic kidney disease (non-dialysis, non-transplant) and co-morbidities, caregivers, and health care providers in Alberta were invited to participate. Participants were recruited through various methods, including in-clinic posters and web-based posts. We assessed responses using descriptive and non-parametric analyses (e.g., Mann–Whitney *U*-test).

**Results:**

Ninety-seven eligible individuals completed the survey; 24 patients, 12 caregivers, and 61 health care providers. Caregivers rated PC-IC significantly lower than patients (overall score: 3.36/5 and 3.91/5, respectively, *p* < 0.05) and health care providers rated PC-IC moderately (3.56/5). The lowest scored domain was care coordination amongst patients and caregivers (3.43/5 and 3/5, respectively, *p* < 0.05) and regional health care laws/regulations amongst health care providers (2.94/5).

**Conclusion:**

Survey respondents recognized that the overall delivery of PC-IC is not optimal and identified key areas to address including improving care coordination (e.g., communication between providers) and tackling regional health care laws/regulations (e.g., funding models). Our study highlights the need for further exploration regarding why PC-IC is perceived as suboptimal, particularly among subgroups, and how it can be improved.

## 1 Introduction

Chronic kidney disease (CKD) affects approximately 13% of the global population (1), including roughly 4 million Canadians (2). Furthermore, it is one of the few non-communicable diseases associated with increasing mortality rates over the past twenty years (3). In Canada, health care costs for individuals living with CKD amount to more than $40 billion per year, including costs related to dialysis and transplantation (2). The high rates of CKD and comorbidity result in large economic and personal burdens for those affected due to reduced quality of life, frequent appointments and hospitalizations, increased need for help with care, and reduced life expectancy (4, 5).

Amongst people with mild to moderate CKD (Category G1–G3), there is a high burden of multimorbidity where a quarter of this population live with three or more additional comorbidities (4). Care for this early-stage population is mainly managed in primary care settings (6, 7). Multidisciplinary teams with relevant specialists (e.g., nephrologists, endocrinologists) and allied health providers (e.g., dietitians) in a single clinic are often only available once their CKD progresses (8–10). As a result, patients are required to coordinate numerous interactions with various health care providers and organizations, which can result in fragmented care (11, 12). Consequent lifestyle changes and declining health not only impact those diagnosed, but also people who are close to them and importantly, those who are involved in their care, such as family caregivers (13).

Person-centered integrated care (PC-IC) is an approach to care delivery that intentionally incorporates the perspectives of individuals living with chronic diseases, their caregivers, and communities from the early onset of disease (14). PC-IC also places a focus on supporting patients to participate in shared-decision making with health care providers involved in managing and delivering their care, while considering the continuum of care across different settings (e.g., primary and specialty care) to ensure consistent health promotion, diagnosis, treatment, and disease-management (14, 15).

PC-IC has been shown to improve health outcomes for individuals with chronic conditions such as diabetes mellitus type 2, chronic respiratory diseases, cardiovascular disease, and kidney failure (16–18). However, evidence regarding the provision of PC-IC for patients with early-stage CKD and co-morbidities and the resulting health and system outcomes is limited. In addition, the measurement of PC-IC delivery has been focused on health administrators and health care providers, thus missing important perspectives of patients and their caregivers.

In this study, we assessed the delivery of PC-IC for people living with mild to moderate CKD and multimorbidity, from the perspectives of patients, caregivers, and health care providers. Our study represents the first phase of a larger project examining gaps in care and opportunities to improve the delivery of PC-IC at the primary care-nephrology care interface in Alberta.

## 2 Materials and methods

### 2.1 Study design and setting

We conducted a cross-sectional study using an online survey to measure the perceived level of PC-IC for people with mild to moderate CKD and comorbidities. The study was approved by the University of Calgary Conjoint Health Research Ethics Board (REB22-1712).

At the time of this study, the provincial delivery system in Alberta, was divided into five health zones that serve remote, rural, and urban jurisdictions and support care to marginalized groups. Within these zones, two provide clinical services to people followed by nephrologists. Primary Care Networks provide care through a team-based model (i.e., primary care physicians and allied health providers) that can be centralized within one clinic or non-centralized across community clinics (19, 20). Payment methods include fee-for-service and alternate relationship plans (e.g., salary, capitation) (21). Alberta Kidney Care and primary care clinics vary in their size, remuneration, available resources, and staff composition (e.g., access to multidisciplinary team).

While there is no current integrated care model in place, there are strategies in the province aimed to improve CKD care across the primary and nephrology care settings, including My Kidneys My Health self-management website (22–30); CKD Pathway to identify, diagnose, and manage CKD (31–34); Kidney Check screening initiative (35); Kidney Failure Risk approach to stratify patients to appropriate care (36), and nephrology eReferral (37) to manage care in remote and rural areas.

### 2.2 Sample and recruitment

Eligible individuals included adults over the age of 18 years who were living in Alberta, Canada, and self-identified as: a patient with mild to moderate CKD (Category G1–G3) with one or more comorbidities (e.g., diabetes, depression, heart failure, hypertension, peripheral vascular disease); a caregiver for someone with mild to moderate CKD; or a health care provider for this population (i.e., primary care physician, nephrologist, allied health professional).

A range of methods were used to recruit participants, including web postings, social media posts, emails to participants from prior research who consented to being contacted for future studies, newsletters, and snowball sampling (i.e., participants identified other potential people). Posters and postcards were also printed and circulated in-person within various primary care and nephrology clinics in Alberta. Interested individuals could access the survey through a link and/or QR code via these methods. Recruitment materials also included the telephone number for a study team member (SJ), should they be interested in completing the survey over the telephone rather than online. Study details and consent information were described on the first page of the survey. Respondents implied informed consent through completion of the survey. Individuals were screened for eligibility through self-identified demographic criteria (e.g., age, geographic location, clinic setting, level of CKD, and comorbidities). Only eligible individuals could proceed through the survey.

### 2.3 Data collection

**Figure 1:**
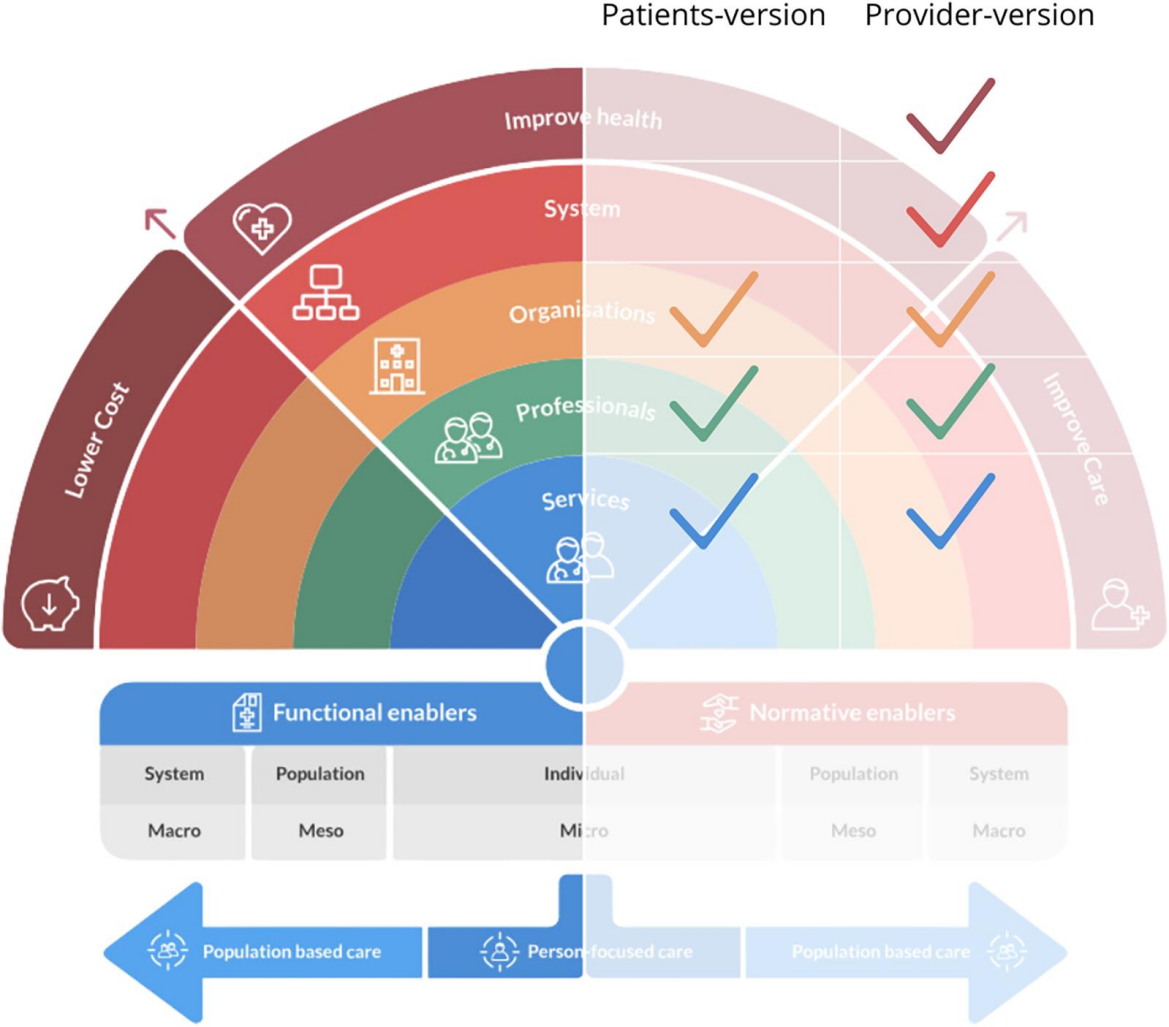
RMIC framework. The Rainbow Model for Integrated Care by P.P. Valentijn, 2015. Copyright 2017 by Essenburgh Group, Harderwijk, The Netherlands. Reproduced with permission (15).

The survey was available in English and included questions about respondent characteristics and questions from the Rainbow Model of Integrated Care Measurement Tool (RMIC-MT) (38). The RMIC-MT is a validated survey based on the RMIC framework that rates the level of PC-IC from the perspectives of health care providers, patients, and caregivers (39). Distinct surveys were used for patients/caregivers and health care providers (Figure 1). The patient/caregiver survey included 16 items categorized into five domains (person-centered care, kidney care coordination, team-based care, care coordination, & access). Patient/caregiver questions also addressed perceived health status, comorbidities, and experiences and interactions with health care providers (specialist and primary care settings) within the last 4 months. The health care provider survey included 16 items categorized into nine domains (person-centered care, community-centeredness, internal care coordination, external care coordination, regional partnerships, regional health care laws and regulations, impact, sharing data, & culture). Health care providers were asked additional questions about their clinic setting and position. For both surveys, respondents were asked to answer the RMIC-MT domain questions using a 5-point Likert scale (i.e., agreement scale: 1 = Strongly disagree and 5 = Strongly agree; frequency scale: 1 = Never and 5 = Always) or categorical, close-ended questions. All respondents were asked to respond to a final open-ended question asking them to comment on care coordination. A forced response approach (i.e., respondents had to answer each question before they could proceed to the next question) was used for the RMIC-MT questions (40).

### 2.4 Analysis

The online survey was available from May to December 2023. Only surveys with all RMIC-MT questions answered were included in our data analysis. Descriptive statistics (means, standard deviations, frequencies, and percentages) were calculated to summarize respondent characteristics and RMIC-MT domain scores. For the 5-point Likert-scale items, weighted mean scores were computed for each RMIC-MT domain and the RMIC-MT overall score, with higher scores reflecting better perceived overall PC-IC. Internal consistency of the RMIC-MT domains was assessed using Cronbach's alpha. Comparative analysis was used to identify statistically significant differences within and between groups (patients/caregivers and health care providers). Due to small sample sizes, particularly in the caregiver subgroup, formal hypothesis testing was limited. Instead, descriptive comparisons were used to explore differences in RMIC-MT overall scores across subgroups (e.g., gender, age, and geographic region). Where appropriate, non-parametric methods (e.g., Mann–Whitney *U*-test) were considered to support exploratory analysis. Between-group comparisons (patients/caregivers vs. health care providers) were restricted to the overall RMIC-MT score, as the domains differ between the two survey versions. Relevant open-ended responses were reviewed and categorized based on the RMIC-MT domains to enrich the quantitative findings.

## 3 Results

### 3.1 Respondent characteristics

Of the 110 individuals who initiated the survey, 97 (88%) completed all RMIC-MT questions and were included in the data analysis. Respondents included 24 patients (25%), 12 caregivers (12%), and 61 health care providers (63%).

Table 1 summarizes the characteristics of patients and caregivers. Of patient respondents indicating their gender, half were men (46%, *n* = 11) and half were women (46%, *n* = 11). More women (75%, *n* = 9) caregivers participated than men (25%, *n* = 3), and the majority were under the age of 35 years (67%, *n* = 8). Most patient respondents were either less than 35 years old (42%, *n* = 10) or over 56 years old (42%, *n* = 10) and had an eGFR greater than 30 ml/min/1.73 m^2^ (67%, *n* = 16). The most commonly reported comorbidities among patients were high blood pressure (hypertension), depression, peripheral vascular disease, and diabetes. Half of patient respondents lived in large metropolitan or metro centers (population >500,000; 50%, *n* = 12), while half of caregivers lived in large urban population areas (population 100,000 to 499,999; 50%, *n* = 6).

**Table 1:** Patient & caregiver characteristics.

Type	Patients (*n* = 24) *n* (%)	Caregivers (*n* = 12) *n* (%)
Age		
<35	10 (42%)	8 (67%)
36–55	4 (17%)	2 (17%)
>56	10 (42%)	2 (17%)
Sex		
Female	11 (46%)	9 (75%)
Male	11 (46%)	3 (25%)
Prefer not to answer	1 (4%)	
No response	1 (4%)	
Gender		
Woman	11 (46%)	9 (75%)
Man	11 (46%)	3 (25%)
Other	1 (4%)	
No response	1 (4%)	
Geography		
Large metropolitan (>500,000)	12 (50%)	4 (33%)
Large urban population (100,000 to 499,999)	4 (17%)	6 (50%)
Medium rural population (30,00 to 99,999)	1 (4%)	1 (8%)
Small rural population (1,000 to 29,999)	3 (13%)	1 (8%)
No response	4 (17%)	
Ethnicity		
White	16 (67%)	9 (69%)
Indigenous	4 (17%)	3 (31%)
Prefer not to answer	1 (4%)	
No response	3 (13%)	
Household income		
$30,000–$49,999	1 (4%)	1 (8%)
$50,000–$69,999	5 (21%)	1 (8%)
$70,000–$99,999	3 (13%)	6 (50%)
$100,000–$149,999	5 (21%)	2 (17%)
$150,000 or more	6 (25%)	1 (8%)
Prefer not to answer	3 (13%)	1 (8%)
No response	1 (4%)	
Kidney Function		
Less than 15% (eGFR 15 ml/min/1.73 m^2^)	2 (8%)	
15% to 29% (eGFR 15–29 ml/min/1.73 m^2^)	3 (13%)	
30% to 60% (eGFR 30–60 ml/min/1.73 m^2^)	6 (25%)	
Greater than 60% (eGFR 60 ml/min/1.73 m^2^)	10 (42%)	
I don’t know	2 (8%)	
No response	1 (4%)	
Other conditions		
High blood pressure	13 (54%)	
Depression	5 (21%)	
Peripheral vascular disease	5 (21%)	
Diabetes	1 (4%)	
Visits to kidney doctor		
1 time a year	5 (21%)	
2–3 times a year	6 (25%)	
4–5 times a year	7 (29%)	
6 or more times a year	4 (17%)	
I do not see a kidney doctor	1 (4%)	
No response	1 (4%)	
Years since diagnosis		
Less than 5 years	14 (58%)	
6–10 years	4 (17%)	
11 years or more	5 (21%)	
No response	1 (4%)	

Table 2 summarizes the characteristics of health care providers. There were 23 nephrologists (38%), 21 primary care physicians (34%), 8 nurses (13%), 3 pharmacists (5%), and 6 categorized as “other/unspecified” (10%). Most respondents were either less than 35 years old (39%, *n* = 24) or between 36 and 55 years old (44%, *n* = 27), and roughly half were women (48%, *n* = 29). Over half worked in a large metropolitan or large urban population area (38%, *n* = 23 & 21%, *n* = 13, respectively). Additionally, approximately half worked in a general nephrology setting (48%, *n* = 29) and most had between zero and ten years of experience (59%, *n* = 36).

**Table 2:** Health care provider characteristics.

Type	Health care provider (*n* = 61) *n* (%)
Age	
<35	24 (39%)
36–55	27 (44%)
>56	5 (8%)
No response	5 (8%)
Sex	
Female	29 (48%)
Male	26 (43%)
Prefer not to answer	1 (2%)
No response	5 (8%)
Gender	
Woman	29 (48%)
Man	26 (43%)
Prefer not to answer	1 (2%)
No response	5 (8%)
Geography	
Large metropolitan (>500,000)	23 (38%)
Large urban population (100,000 to 499,999)	13 (21%)
Medium rural population (30,00 to 99,999)	8 (13%)
Small rural population (1,000 to 29,999)	12 (20%)
No response	5 (8%)
Ethnicity	
White	35 (57%)
Indigenous	2 (3%)
Black	11 (18%)
East Asian	3 (5%)
Middle Eastern or North African	1 (2%)
South Asian	1 (2%)
Southeast Asian	2 (3%)
Prefer not to answer	1 (2%)
No response	5 (8%)
Role	
Nephrologist	23 (38%)
Primary care physician	21 (34%)
Nurse	8 (13%)
Pharmacist	3 (5%)
Other	1 (2%)
No response	5 (8%)
Years of practice	
0 to 5 years	17 (28%)
6 to 10 years	19 (31%)
11 to 15 years	8 (13%)
16+ years	12 (20%)
No response	5 (8%)
Setting	
General nephrology	29 (48%)
Kidney care clinic	13 (21%)
Primary care practice	8 (13%)
Other^a^	6 (10%)
No response	5 (8%)

^a^ Kidney care clinic and general nephrology (*n* = 1); Community pharmacy (*n* = 2); Transplant clinic (*n* = 1); Both general nephrology and kidney care clinic (*n* = 1); Inpatient (*n* = 1).

### 3.2 Rainbow model of integrated care measurement tool (RMIC-MT) scores

#### 3.2.1 RMIC-MT overall scores

**Figure 2:**
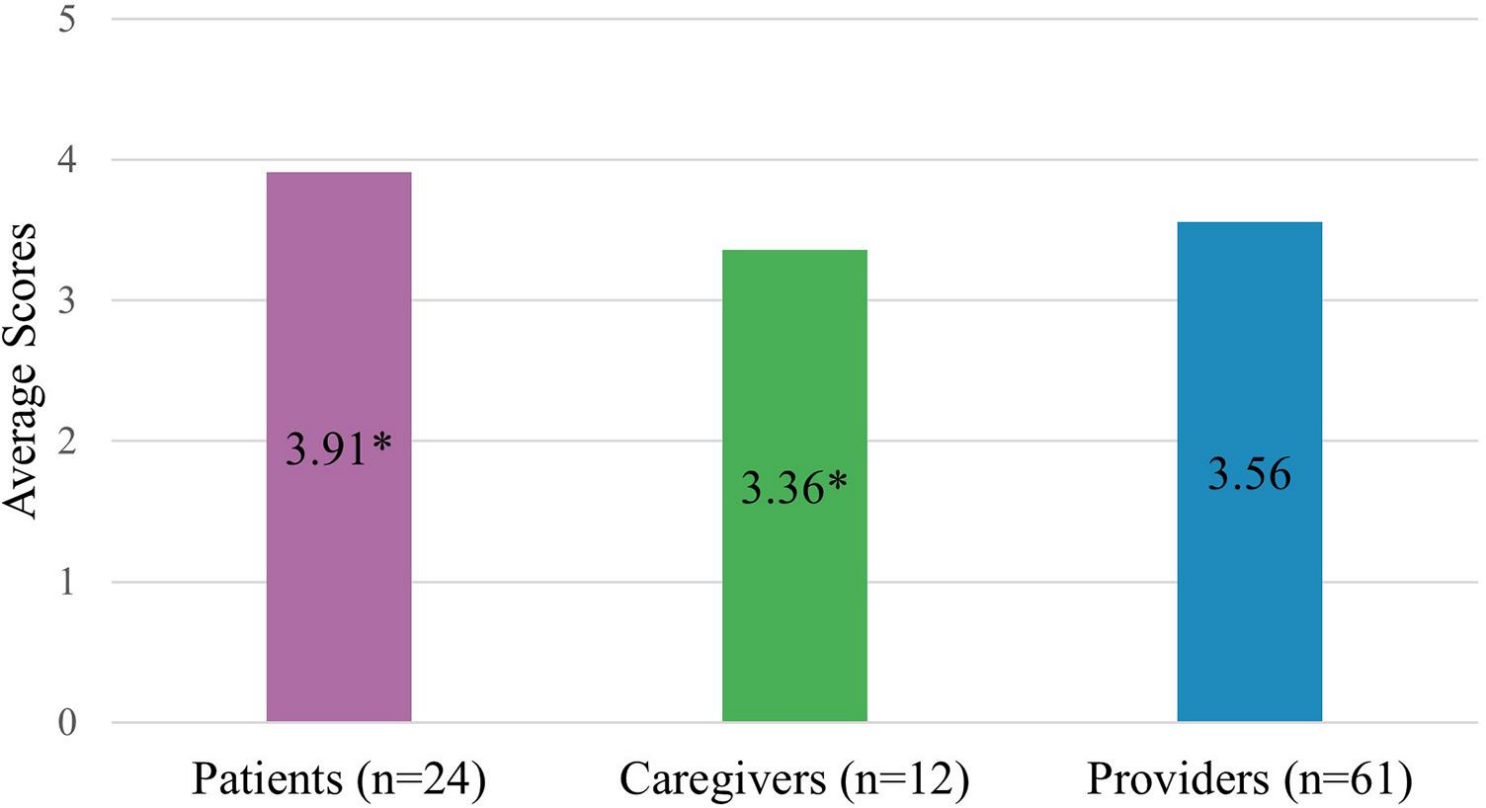
Overall RMIC-MT integrated care scores for all groups. * Indicates a statistically significant difference (*p* < 0.05).

Caregivers and health care providers rated overall PC-IC lower (RMIC-MT overall score; 3.36/5 and 3.56/5, respectively) as compared to patients (3.91/5; Figure 2), with a statistically significant difference only between caregivers and patients (Figure 2; *p* *<* *0.05*). In addition, health care providers who identified as women rated overall PC-IC significantly lower than men (RMIC-MT overall score; 3.30/5 and 3.70/5 respectively; *p* *<* *0.05*). In contrast, patients and caregivers who were women rated PC-IC higher than men counterparts 3.85/5 and 3.60/5, respectively (*p* *<* *0.05*). Primary care physicians rated PC-IC significantly lower (3.22/5) than nephrologists (3.71/5; *p* *<* *0.05*).

#### 3.2.2 RMIC-MT domain scores

**Figure 3:**
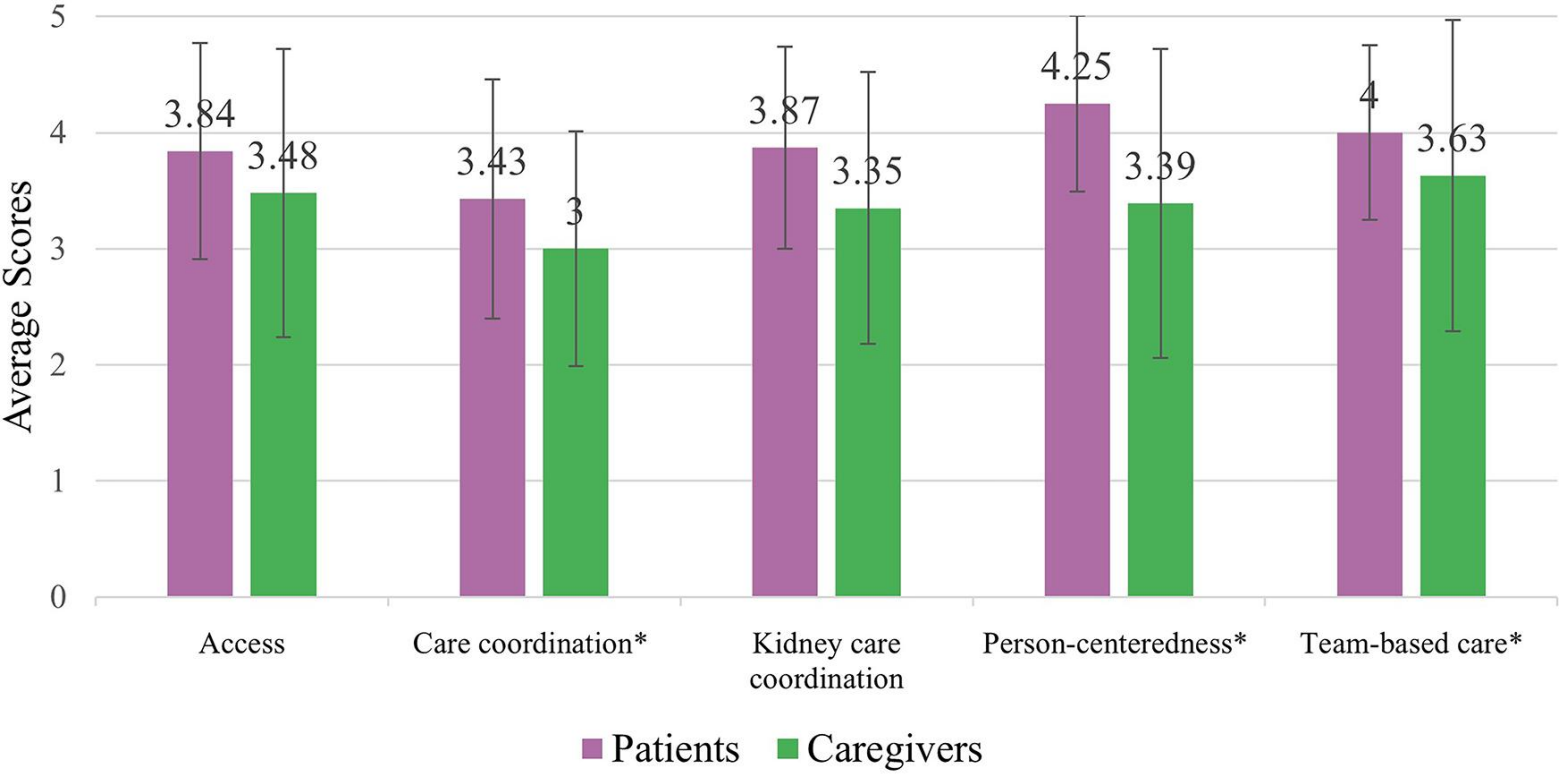
Patient (*n* = 24) & caregiver (*n* = 12) RMIC-MT domain scores. * Indicates a statistically significant difference (*p* < 0.05).

Among patient/caregiver RMIC-MT domains (i.e., access, care coordination, kidney care coordination, person-centeredness, and team-based care), care coordination was scored the lowest by both patients and caregivers, with caregivers scoring this domain significantly lower than patients (mean scores 3.43/5 and 3.00/5, respectively*, p* *<* *0.05*; Figure 3 & Table 3). Patients reported higher mean scores than caregivers for person-centeredness of care (4.25/5 and 3.39/5 respectively, *p* *<* *0.05*) and team-based care (patients: 4.00/5, caregivers: 3.63/5, *p* *<* *0.05*). In open-text responses, one patient responded;

**Table 3:** RMIC-MT patients & caregiver domains, domain descriptions, and mean scores.

Domain	Definition	Patient mean score (SD)	Caregiver mean score (SD)
Access	Accessibility of care (e.g., location, booking of appointments, results, different providers)	3.84 (0.93)	3.48 (1.24)
Care coordination	The cooperation between kidney care team and other health care providers visited outside of kidney care team	3.43 (1.03)*	3.00 (1.01)*
Kidney care coordination	Coordination of kidney care team (e.g., nephrologist, family doctor, nurse)	3.87 (0.87)	3.35 (1.17)
Person-centeredness	How the patient is cared for by their team (e.g., nephrologist, nurse, other care provider)	4.25 (0.76)*	3.39 (1.33)*
Team-based care	How the care providers in the clinic work together	4.00 (0.75)*	3.63 (1.34)*

* *p*-value <0.05.

“I am very pleased with my gastroenterologist, internal medicine doctor, and nephrologist. They work as a team. Netcare [provincial electronic health record system] makes it easy to bring up my medical history. Other areas are not so good (…). Bloodwork wait time was horrendous, even with an appointment you have to wait 45 min to an hour. (…) I have 7 specialists and a family doctor. You get medical fatigue”.—Patient

In contrast, one caregiver wrote;

“I think as a caregiver, I’m not asked questions in our visits. Everything is applied to him [patient]. And I think he's not completely honest about his symptoms or how he really is feeling. Also, being a caregiver I feel maybe there should be more support for us as we live with it 24/7”.—Caregiver

**Figure 4:**
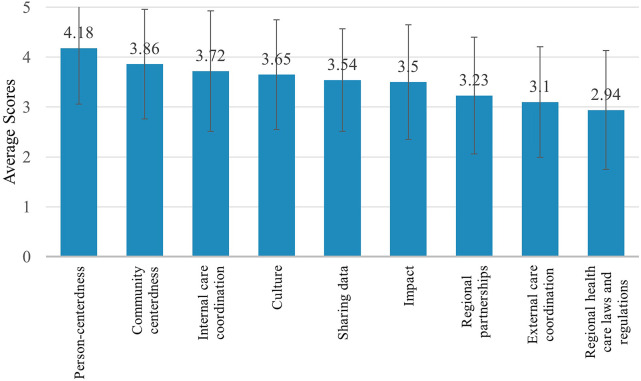
Health care provider (*n* = 61) RMIC-MT domain scores.

For the health care provider RMIC-MT domains (i.e., community centeredness, culture, external care coordination, impact, internal care coordination, person-centeredness, regional health care laws and regulations, regional partnerships, and sharing data), health care providers scored person-centeredness highest (4.18/5). External care coordination (3.10/5) and regional health care laws and regulations (2.94/5) were scored the lowest (Figure 4 & Table 4). Open text responses from health care providers included statements such as;

**Table 4:** RMIC-MT health care providers domains, domain descriptions, and mean scores.

Domain	Definition	Mean score (SD)
Community-centeredness	The extent to which the health of a neighbourhood/region is central	3.86 (1.10)
Culture	The extent to which the partners help and support each other within the clinic	3.65 (1.10)
External care coordination	The extent of the cooperation between the clinic and other care providers outside the clinic (e.g., cardiologist, family doctor)	3.10 (1.11)
Impact	The extent to which the results are monitored and followed up	3.50 (1.15)
Internal care coordination	The extent of the clinic's ability to coordinate care with external care providers, clinics, and/or hospitals	3.72 (1.21)
Person-centeredness	The extent to which the needs and capabilities of the patients are central	4.18 (1.12)
Regional healthcare laws and regulations	The extent to which legislation and regulations stimulate cooperation	2.94 (1.19)
Regional partnerships	The extent to which the various care providers work together	3.23 (1.17)
Sharing data	The extent to which data is shared between the various care providers	3.54 (1.03)

“It is easier to coordinate care with a shared EMR [electronic medical record]”, and “I realize there is room for improvements with staff relationships and leadership, which would in turn improve patient care in relation to busy or stressful work loads”.—Health care provider

No statistically significant associations were observed between domain scores and length of time in practice, age, geography, or clinical setting among health care providers.

## 4 Discussion

This cross-sectional survey measured PC-IC for people with mild to moderate CKD and multimorbidity. To our knowledge this is the first study to systematically measure PC-IC for this population from not only perspectives of health care providers, but patients and caregivers. Overall PC-IC was measured and several areas for improvement were identified, as well as differences in perspectives amongst participants. For example, there were statistically significant differences in overall PC-IC scores based on gender and role. Gender can influence how patients, caregivers, and health care providers interact, their values, and their power within social contexts (41). Further qualitative research is being conducted to understand the gaps in PC-IC delivery, as well as exploration of the trends identified by gender.

We found that while patients rated overall PC-IC favourably, caregivers and health care providers rated it lower, with a statistically significant difference between patients and caregivers in three domains. Team-based care (how health care providers in a clinic work together) was perceived differently by caregivers and patients, with caregivers rating it significantly lower, highlighting a potential missed opportunity for integrated care provision that also attends to caregiver needs. Care coordination (the cooperation between the kidney care team and other health care providers outside of the kidney care team) was scored the lowest by patients and caregivers and identified as a key area for improvement. Similarly, health care providers scored external care coordination (cooperation between the clinic and other care providers outside of the clinic) as the second lowest domain, suggesting room for improvement for all participant groups on this domain of PC-IC. In addition, regional health care laws/regulations (legislation to support health care providers to work cooperatively) was ranked the lowest by health care providers. Person-centeredness (needs of patients are central to their care by team) was rated higher by patients and health care providers than caregivers. Although assessed domains were different between patients/caregivers and health care providers, health care providers rated areas such as care coordination, person-centeredness, and overall PC-IC delivery in between those of patients and caregivers.

Caregivers play a key role in supporting patients with CKD and multimorbidity and can provide a unique perspective regarding PC-IC provision. There has been an increase of “outsourcing” care to informal caregivers, such as family members, who will often act as coordinators (42, 43). Our study found that caregivers rated overall PC-IC lower than patients and health care providers, and that caregivers scored every RMIC-MT domain lower that patients. Whereas caregivers scored person-centeredness significantly lower than patients, health care providers scored it the highest out of all the domains. Open-ended responses provide some insight into the potential reasons for a mismatch in scores between health care providers and caregivers. Specifically, caregivers' frustration with lack of integration into clinical interactions or concerns that the person they care for may not engage in truthful dialogue with the health care team. Scholars have identified similar challenges regarding health care provider approaches to managing patient and caregiver interactions and have found that shared goals or knowledge with both patients and caregivers is not sufficient (44). Instead, dedicated resources or support can help to improve caregiver perception, as well as patient outcomes (43). The patient-family caregiver interaction can be difficult for health care providers to navigate, especially as patients age and cognition may decline; however, it is extremely important to address (44). Caregivers actively engage in a patient's health care, and collaborative discussions that include the caregiver, compared to individual discussions between provider and patient, can allow for increased participation by the caregiver (44).

Not only was there a significant difference in caregiver and patient scores for care coordination, but as a domain it was scored the lowest by these groups. This suggests the need for cooperation and care coordination between the kidney care team and other care providers outside of the kidney care team (e.g., primary care physicians, pharmacists). Caregivers scored this category significantly lower than patients, and this may be because caregivers are often, but not always, responsible for instrumental support, such as managing the patient's care and appointments. Similar to the care coordination domain for patients and caregivers, external care coordination received low scores by health care providers. Overall, all groups indicated that care coordination was lacking between health care providers across teams in different settings (i.e., between nephrology and primary care clinics). One study assessing the delivery of coordinated care for people with advanced CKD in the Canadian province of Ontario found inconsistencies in the perception of how care is coordinated (45), demonstrating that the results of this study are not isolated to Alberta.

Our caregiver findings are important, as research has shown that caregivers are increasingly taking on roles to support PC-IC that were previously assumed by health care providers, such as system navigators and coordinators (46). The increased burden on caregivers is demonstrated through their significantly lower rating of the team-based care domain than patients. Team-based care measures how care providers in the clinic work together, and as caregivers take on increased responsibilities and roles as informal system navigators, they may have unique insight how health care teams work together. Recognizing caregivers as key members of the care team and acknowledging their needs related to PC-IC can provide beneficial outcomes for patients and the health system, while decreasing caregiver burden and stress (13, 47). Further research and support is needed in this area, recognizing caregivers as care coordinators, while they navigate the increasingly complex care system. Opportunities to do this may include increased recognition for caregivers as coordinators through the lens of health care providers, the provision of online tools for caregivers specifically, or increased communication tools between hospitals and home (42, 48).

Health care providers in Alberta rated regional health care laws and regulations as the lowest RMIC-MT domain indicating that there are worries or frustrations within Alberta, which is also a sentiment expressed nationally and with a focus on primary care (49, 50). Although no specific regulatory barriers were specifically referenced in the survey or mentioned by health care providers in the open-text response, possible factors that could have influenced this domain's score include the highly decentralised nature of Canada's health care system and financing of health services. Canada's universal health care system is governed by the Canada Health Act (1984), which means that health care services are delivered by the individual provinces and territories, but each receives federal funding through block grants for meeting certain criteria (49, 51). Service delivery is therefore highly decentralised as each province and territory retains the ability to manage the day-to-day delivery and administration of care (49). Despite their individual jurisdictions, provinces and territories share similar challenges of staff shortages (nurses, physicians, specialists), resource constraints, and funding models, and Alberta is no exception (49, 50, 52). Financing may also play a large role through how physicians are paid (e.g., fee-for-service) and the variation in what is covered by the government (i.e., public services including medically necessary physician services), what is covered by a mix of public and private or out-of-pocket payments (i.e., outpatient prescription drugs), and finally, what is fully privately financed (i.e., outpatient physiotherapy) (51).

There are notable differences between our results and international benchmarks. Our results contrast with those of a validation study of the RMIC-MT involving 8,421 health care providers and 30,788 patients with CKD from 19 countries (39). The RMIC-MT domains of access, care coordination, and kidney care coordination were scored lower in our patient respondents than among patients with CKD in the international study. Additionally, health care provider respondents scored kidney care coordination lower in Alberta compared to international respondents and differed notably in their scores for the regional health care laws and regulations domain. The only domain that was scored higher in Alberta than in the international study was patient respondents' rating of person-centeredness. Despite Canada's provision of public health care our results suggest that current PC-IC in Alberta falls short of international benchmarks with potential to address areas of improvement in the provision of PC-IC for people living with CKD and comorbidities. Compared to other nations, Canada faces several challenges, such as its immense geography making health system provision to rural or remote communities difficult, long wait times, and varied access to services outside of hospitals (51, 53). The differences in PC-IC domain perception provides an opportunity for Alberta to observe models of care and PC-IC provision in other contexts and adapt them to the local and regional settings with a specific focus on domains of concern, such as those previously mentioned.

### 4.1 Strengths and limitations

We used an internationally validated measurement tool that has been designed for different populations (patients/caregivers and health care providers) and tested in various settings and jurisdictions. We were able to collect data from not only from physicians, but other health care providers, patients, and caregivers who are commonly underrepresented in research looking at PC-IC delivery. Our study is strengthened by the inclusion of socio-demographically and clinically diverse respondents. Respondents resided in both rural and urban settings, which are important considerations when examining coordination of care and access to health care providers.

Our study includes some limitations. The survey was voluntary and included self-reported demographic questions (e.g., CKD status) which may have resulted in an increased risk of nonrespondent and misclassification bias, respectively. Although 110 interested individuals initiated the online survey, 97 fully completed the survey. This may be due to the forced response survey design, which may have led to attrition, as well as affected data quality through respondent bias (54). We believe the impact was minimal, given that there was a small percentage of respondents who did not complete the survey. The majority of our caregiver respondents were women, which is reflective of the typical caregiving role distribution for supporting individuals with chronic diseases (55, 56). While the caregiver respondents were a smaller group which impacts the generalizability of their results, their inclusion allowed for another viewpoint of care not commonly considered. We also had a lower number of allied health respondents, which may reflect views and experiences of these groups are underrepresented. Finally, our survey was completed in English only, in only one province in Canada, and at one point in time which may limit the generalizability and types of respondents included; however, the challenges faced by provinces in administering Canada's universal, publicly funded health care system are similar for people with CKD and multimorbidity (e.g., inequitable access to services, fiscal constraints, etc.) regardless of geographic location (51).

## 5 Conclusion

In conclusion, research focused on PC-IC in kidney care for patients with early-stage CKD has been limited. Patients, caregivers, and health care providers recognize that the overall delivery of PC-IC is not optimal and have identified key areas that need to be addressed including improving care coordination, team based and person-centeredness care, and tackling regional health care laws/regulations. Our study highlights the need for further exploration regarding why PC-IC is suboptimal and how integrated care can be improved.

## Data Availability

The datasets presented in this article are not readily available because in compliance with our research ethics board, data are not available for use by other researchers. Questions can be directed to the corresponding author (MD). Requests to access the datasets should be directed to donaldm@ucalgary.ca.
